# All Three Rows of Outer Hair Cells Are Required for Cochlear Amplification

**DOI:** 10.1155/2015/727434

**Published:** 2015-07-30

**Authors:** Michio Murakoshi, Sho Suzuki, Hiroshi Wada

**Affiliations:** ^1^Department of Mechanical Engineering, Kagoshima University, 1-21-40 Korimoto, Kagoshima 890-0065, Japan; ^2^PRESTO, JST, 4-1-8 Honcho, Kawaguchi 332-0012, Japan; ^3^Department of Bioengineering and Robotics, Tohoku University, 6-6-01 Aoba-yama, Sendai 980-8579, Japan; ^4^Department of Intelligent Information Systems, Faculty of Science and Technology, Tohoku Bunka Gakuen University, 6-45-1 Kunimi, Sendai 981-8551, Japan

## Abstract

In the mammalian auditory system, the three rows of outer hair cells (OHCs) located in the cochlea are thought to increase the displacement amplitude of the organ of Corti. This cochlear amplification is thought to contribute to the high sensitivity, wide dynamic range, and sharp frequency selectivity of the hearing system. Recent studies have shown that traumatic stimuli, such as noise exposure and ototoxic acid, cause functional loss of OHCs in one, two, or all three rows. However, the degree of decrease in cochlear amplification caused by such functional losses remains unclear. In the present study, a finite element model of a cross section of the gerbil cochlea was constructed. Then, to determine effects of the functional losses of OHCs on the cochlear amplification, changes in the displacement amplitude of the basilar membrane (BM) due to the functional losses of OHCs were calculated. Results showed that the displacement amplitude of the BM decreases significantly when a single row of OHCs lost its function, suggesting that all three rows of OHCs are required for cochlear amplification.

## 1. Introduction

**Figure 1:**
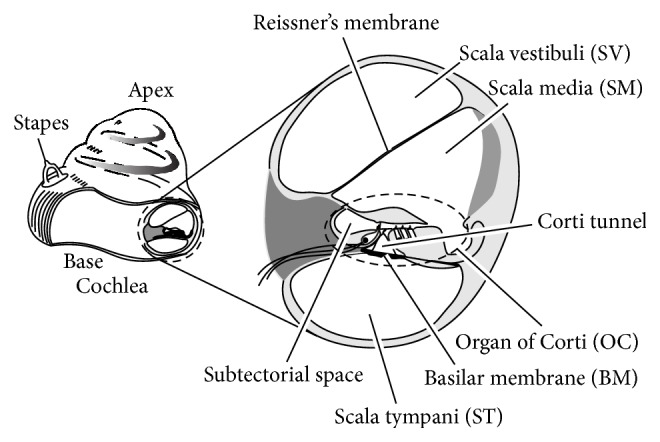
Schematic of the gerbil cochlea and its cross section. The three fluid-filled ducts named scala vestibuli (SV), scala media (SM), and scala tympani (ST) are separated from each other by Reissner's membrane and the basilar membrane (BM). The organ of Corti (OC) contains sensory cells that detect sound.

**Figure 2:**
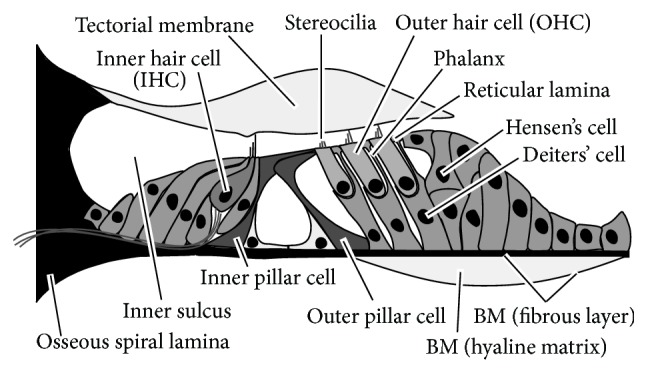
Schematic of structure of the OC. The OC contains two types of sensory cells, that is, inner hair cells (IHCs) and outer hair cells (OHCs). The tectorial membrane (TM) is an extracellular matrix and covers the OC. The BM is located beneath the OC, which is composed of the fibrous layer and the hyaline matrix. Scale bar equals 50 *μ*m.

Sounds are converted to mechanical vibration at the tympanic membrane and this vibration is transmitted to the cochlea in the inner ear. The cochlea is a fluid-filled duct coiled like a snail shell.  Figure 1 shows a schematic of the cross section of the cochlea, which is divided into three compartments by longitudinal membranes. The upper membrane, Reissner's membrane, separates the scala vestibuli (SV) from the scala media (SM). The lower membrane, the basilar membrane (BM), separates the scala tympani (ST) from the SM. On the BM, there is a sensory organ of hearing called the organ of Corti (OC), which has two types of sensory cells: inner hair cells (IHCs) in a single row and outer hair cells (OHCs) in three rows.  Figure 2 shows a schematic of the OC. Since the OC sits on the BM, it vibrates in synchronization with the BM vibration and this OC vibration induces shear motion between the tectorial membrane (TM) and the reticular lamina (RL). Due to this shear motion, the stereocilia of both types of hair cells are bent, and thus ionic current flows into the IHCs and OHCs, changing their membrane potentials. Regarding the IHCs, cell activation caused by stereocilia deflection results in the generation of impulses to the auditory afferent nerve via synaptic connections, leading to recognition of sound. On the other hand, OHCs contract and elongate in response to their changes in membrane potential. Because of this OHC function, OHCs are thought to increase the displacement amplitude of the OC. This process is known as a cochlear amplification, contributing to the high sensitivity, wide dynamic range, and sharp frequency selectivity of our hearing.

Recent studies have shown that noise exposure, ototoxic acid, aging, and so forth cause the functional loss of OHCs, resulting in hearing loss. However, the degree of decrease in cochlear amplification when the OHCs in one, two, or all three rows have lost their function remains unclear. Since the OC is vulnerable and sound-induced displacement amplitude of the OC is quite small, measurement of the dynamic behavior of the OC is difficult. Therefore, analysis using the finite element method (FEM) is helpful.

In the present study, a model of the gerbil cochlea including cochlear amplification by OHCs was reconstructed based on a previous model [1]. Then, to determine effects of the OHC functional loss on the cochlear amplification, changes in the BM displacement amplitude due to the functional loss of OHCs in one, two, or all three rows were analyzed by calculation using FEM.

## 2. Modeling

### 2.1. Geometry

#### 2.1.1. Model of OC

In the previous study, an OC model at the basal turn of the gerbil cochlea, which is comprised of a two-dimensional model of the OC and three-dimensional models of the lymph fluid surrounding the OC model, was developed by Andoh et al. [1].

**Figure 3:**
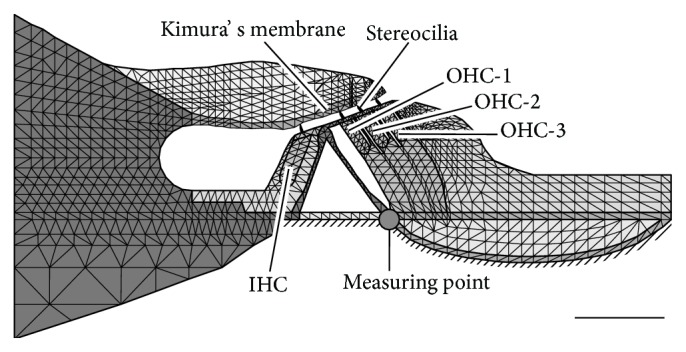
Model of the OC discretized with elastic finite elements. The number of nodes is 1,310 and the number of elements is 2,251. The hatched area indicates the place where initial pressure to this model was applied. The filled circle represents the point where the displacement of the BM was measured. Scale bar equals 50 *μ*m.

The length of the BM of the gerbil is approximately 12 mm [2] and the sound-induced displacement amplitude of the BM in the gerbil cochlea is approximately 5 nm when a pure tone of 80 dB SPL is applied to the ear canal [3]. Due to this small displacement amplitude and the longitudinally extending structure of the OC, it is assumed that the cross section of the OC maintains its plane surface when sound pressure is applied. A two-dimensional model of the OC was therefore constructed under the plane strain condition, as shown in Figure 3. Right and left boundaries of the OC model are fixed. Meshing is done at a subcellular level using triangular elements. The number of nodes and elements are 1,310 and 2,251, respectively. The characteristic frequency (CF), the most effective stimulus frequency, of the present model is 16 kHz.

#### 2.1.2. Models of Lymph Fluid Surrounding OC Model

**Figure 4:**
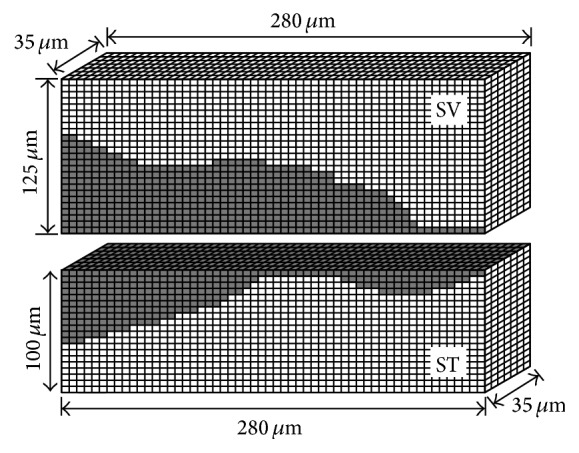
Models of the lymph fluid surrounding the OC model, that is, in the SV and in the ST. Dark area in each model corresponds to the OC. Models of the SV and the ST have 11,200 and 8,000 cubic elements, respectively.

**Figure 5:**
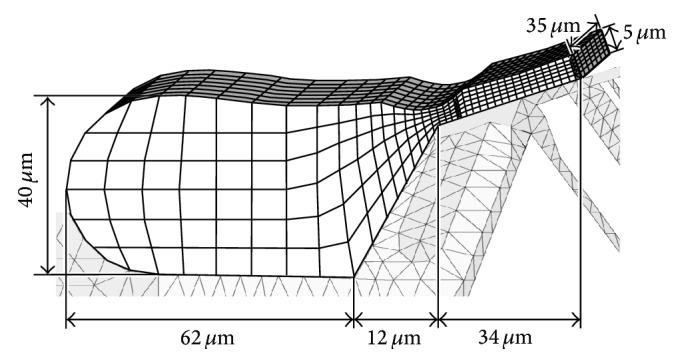
Model of the lymph fluid in the subtectorial space. The subtectorial space is a fluid-filled space below the TM and the extent of the model is from the inner sulcus to the innermost OHC. The number of nodes is 2,128 and the number of elements is 1,554.

To consider the influence of the lymph fluid surrounding the OC model, FEM models of the lymph fluid in the SV, SM, ST, and subtectorial space were constructed, as shown in Figures 4 and 5 [1]. Fluid models are three-dimensional because the lymph fluid moves not only in the radial direction (width direction of the BM) and transversal direction (perpendicular to the BM) but also in the longitudinal direction.

Since Reissner's membrane, which separates the SV and SM, is acoustically transparent and is thought to have little effect on the mechanical functions of the cochlea, the fluid models of the SV and the SM are considered as single compartments in the fluid model of the SV. In consideration of the modiolus and the cochlear wall, the left and right boundaries of SV and ST models are fixed. The top boundary of the SV model and bottom boundary of the ST model are also fixed because it is assumed that the lymph fluid does not move across those boundaries. Meshing of the fluid models is done using 5 *μ*m square cubic elements. The numbers of elements of the SV model and the ST model are 11,200 and 8,000, respectively.

In the subtectorial space model, the intervals of mesh in the cross section are varied to avoid severe distortion of the cubic shape of the element. The intervals of the mesh in the longitudinal direction are 5 *μ*m, the same as those in the SV and ST models. The number of elements of the subtectorial space model is 1,554.

The effect of the mass of the fluid in the subtectorial space, where there is a narrow space between the TM and RL, is assumed to be negligible because the volume of this space is inconsiderable in comparison with those of the SV and SM.

#### 2.1.3. Mechanical Properties

Young's moduli assigned to components of the OC model were determined by Andoh et al. [1] based on the measurement data obtained in previous studies. As shown in Table 1, Young's modulus assigned to the model is 1.0 × 10^4^ Pa at the OHCs [4], 1.0 × 10^7^ Pa at the phalanxes [5], 1.0 × 10^9^ Pa at the pillar cells [6], 1.0 × 10^7^ Pa at the stereocilia [7], and 3.0 × 10^4^ Pa at the TM [8]. Young's moduli of the hyaline matrix in the BM, IHC, and Deiters' cells are assumed to be the same as those of the TM, OHCs, and phalanxes, respectively. Young's modulus for the osseous spiral lamina is assumed to be 2.0 × 10^10^ Pa, which is the same value as that of the cortical bone [9] because both of them are composed of bony material. Young's moduli of the fibrous layer of the BM, Hensen's cells, Kimura's membrane, which is the lower surface of the TM, and the RL are determined to be 1.0 × 10^7^ Pa, 5.0 × 10^3^ Pa, 1.0 × 10^6^ Pa, and 1.0 × 10^9^ Pa, respectively [1].

Poisson's ratio of cells with a relatively small Young's modulus, that is, less than 1.0 × 10^4^ Pa, was assumed to be 0.49 because these portions are thought to be nearly incompressible. On the other hand, those assigned to the other cells and bony structure were assumed to be 0.30, which is commonly used in structural analysis.

### 2.2. Formulation

#### 2.2.1. OC Model

In the OC model, it is assumed that the cross section of the OC maintains its plane surface when external force is applied. Therefore, the model of the OC is formulated under the plane strain condition. The equation of the motion of the structure by the FEM process is the same as that in the previous report [1] represented by the following matrix differential equation:

(1) $$\begin{array}{c} \left[\;M\;\right]\frac{\partial^{2}u}{\partial t^{2}}+\left[\;C\;\right]\frac{\partial u}{\partial t}+\left[\;K\;\right]u=f, \end{array}$$

where [*M*], [*C*], and [*K*] are the mass, damping, and stiffness matrices, respectively, **u** is the structural displacement vector, **f** is the force vector, and *t* is the time. The damping matrix [*C*] is derived from the linear combination of mass and stiffness matrices; that is,

(2) $$\begin{array}{c} \left[\;C\;\right]=\alpha \left[\;M\;\right]+\beta \left[\;K\;\right], \end{array}$$

where *α* and *β* are Rayleigh damping parameters. In this study, *α* and *β* were set to be 1.0 × 10^−40^ s^−1^ and 1.5 × 10^−6^ s, respectively [10]. In the Newmark-*β* method [11], the structural displacement and velocity vectors at the end of a time interval **u** ^*n*+1^ and ∂**u** ^*n*+1^/∂*t* can be expressed in terms of the structural displacement, velocity, and acceleration vectors at the beginning of the time interval **u** ^*n*^, ∂**u** ^*n*^/∂*t*, and ∂^2^ **u** ^*n*^/∂*t* ^2^ by the relations

(3) un+1=un+δt∂un∂t+δt2β∂2un+1∂t2+12−β∂2un∂t2,

(4) ∂un+1∂t=∂un∂t+δt2∂2un+1∂t2+∂2un∂t2,

where *δt* is the time interval and *n* is the time step. In this study, the parameter *β* = 1/4, which yields the constant average acceleration method, was used. Rewriting (3) and (4) lead to

(5) ∂un+1∂t=−∂un∂t+2δtun+1−un,

(6) ∂2un+1∂t2=−∂2un∂t2−4δt∂un∂t+4δt2un+1−un.

Substituting (5) and (6) into (1) at the *n* + 1 step leads to

(7) K+2δtC+4δt2Mun+1=fn+1+M∂2un∂t2+4δt∂un∂t+4δt2un+C∂un∂t+2δtun.

Using (7), **u** ^*n*+1^ can be obtained from the previously determined values of the structural displacement vector **u** ^*n*^ and the known value of the force vector **f** ^*n*+1^.

#### 2.2.2. Fluid Models

Regarding the lymph fluid, the Reynolds number (Re) of fluid flow is defined as

(8) $$\begin{array}{c} Re=\frac{\rho UL}{\mu }, \end{array}$$

where *U* is the approximate maximum fluid velocity in the vicinity of the BM, *L* is the length of the BM, *ρ* is the density of water (1.0 × 10^3^ kg/m^3^), and *μ* is the viscosity of water (1.0 × 10^−3^ Pa·s). As characteristic values are *U* = 1.0 mm/s estimated from experimental data [12] and *L* = 170 *μ*m, Re becomes 0.17. In this range of Reynolds numbers, an incompressible and viscous flow can be assumed. Therefore, an incompressible Navier-Stokes equation is used to analyze the dynamic behavior of the lymph fluid. The incompressible Navier-Stokes equation is as follows:

(9) $$\begin{array}{c} \frac{\partial v}{\partial t}+\left(\;v\cdot \nabla \;\right)v+\frac{1}{\rho }\nabla p_{OC}-\nu \Delta v=0, \end{array}$$

where **v** is the fluid velocity vector, *p*_OC_ is the fluid pressure caused by the OC motion, *ν* is the kinetic viscosity of the fluid, *t* is the time, and the gradient operator ∇ and the Laplacian operator Δ are defined in the following form:

(10) ∇=∂∂x,∂∂y,∂∂z,Δ=∂2∂x2+∂2∂y2+∂2∂z2.

Using the Marker-and-Cell (MAC) method [13], the fluid is decomposed into rectangular parallelepiped cells and the pressure is discretized at the center of each cell. Discretizing the convection term (**v** · Δ)**v** and diffusion term *ν*Δ**v** explicitly and discretizing the pressure term (1/*ρ*)∇*p*_OC_ implicitly in (9), the discrete Navier-Stokes equation is derived as follows:

(11) $$\begin{array}{c} \frac{v^{n+1}-v^{n}}{\delta t}+\left(\;v^{n}\cdot \nabla \;\right)v^{n}+\frac{1}{\rho }\nabla {p_{OC}}^{n+1}-\nu \Delta v^{n}=0, \end{array}$$

where *δt* is the time interval and *n* is the time step. Rewriting (11) leads to

(12) $$\begin{array}{c} v^{n+1}=v^{n}-\delta t\left\{\;\left(\;v^{n}\cdot \nabla \;\right)v^{n}+\frac{1}{\rho }\nabla {p_{OC}}^{n+1}-\nu \Delta v^{n}\;\right\}. \end{array}$$

Considering the divergence of (12) leads to

(13) ∇·vn+1=∇·vn−δt∇·vn·∇vn+1ρ∇pOCn+1−νΔvn.

Following the continuity equation of fluid, ∇·**v** ^*n*+1^ = 0. By contrast, to reduce numerical error, the first term of the right side ∇·**v** ^*n*^ is allowed to remain. Consequently, (13) becomes

(14) $$\begin{array}{c} \Delta {p_{OC}}^{n+1}=\rho \left[\;\frac{1}{\delta t}\nabla \cdot v^{n}-\nabla \cdot \left\{\;\left(\;v^{n}\cdot \nabla \;\right)v^{n}-\nu \Delta v^{n}\;\right\}\;\right]. \end{array}$$

Substituting the known value of fluid velocity vector **v** ^*n*^ into (14), *p*_OC_^*n*+1^ can be obtained and then **v** ^*n*+1^ is obtainable from (12).

#### 2.2.3. Shear Force Exerted on the TM and RL

In the narrow space between the TM and RL, Couette flow is assumed to occur because of the shear motion between them, and the force caused by this shear motion is considered to be exerted on the TM and RL. In this case, the force vectors **f**_TM_ and **f**_RL_ are given by

(15) fTM=μVrelativehATM,

(16) fRL=μVrelativehARL,

where *V*_relative_ is the relative velocity vector of the TM in relation to the RL, *h* is the height of the narrow space between the TM and RL, *A*_TM_ is the area of the undersurface of the TM above the RL, *A*_RL_ is the area of the RL facing the lymph fluid, and *μ* is the viscosity of the lymph fluid.

#### 2.2.4. Force Generated by OHC

Knowledge of the dynamic characteristics of the force generated by the OHC motility in the OC is indispensable to understand the mechanism of the cochlear amplification. The magnitude of the force generated by the OHC has been demonstrated using the isolated OHC [14, 15]. Frank et al. showed that the magnitude of the force generated by OHC motility is at a constant level over the entire audible frequency range [15]. Regarding the phase of the force generated by the OHC relative to the deflection of the hair bundle, a phase delay of 90 degrees is suggested in the high frequency range; this delay is probably resulting from low-pass filtering at the membrane due to membrane resistance and membrane capacitance [16] (see Appendix). Although there are several experimental reports and/or numerical reports, the exact dynamic characteristics of the force generated by the OHC are still controversial.

Using the organotypic culture of the OC, Géléoc et al. reported that the relationship between the displacement amplitude of the stereocilia *x* and the ion current *I*, which flows into the hair cell when the stereocilia are bent statically by a fluid jet, is expressed by the second-order Boltzmann function [17]. In this study, if the above-mentioned experimental results and the CF of the present model, that is, 16 kHz, are taken into account, since the OHC membrane potential is proportional to the transducer current for a given stimulation frequency [18] and since the force generated by the OHC is thought to be proportional to its membrane potential [15], the relationship between the displacement amplitude of the stereocilia *x* and the force generated by OHC *F*_OHC,0_ is assumed to be expressed by the second-order Boltzmann curve:

(17) $$\begin{array}{c} F_{OHC,0}\left(\;x\;\right)=\frac{F_{max}}{1+e^{a_{1}\left(x_{1}-x\right)}\left(1+e^{a_{2}\left(x_{2}-x\right)}\right)}, \end{array}$$

where *F*_max_ is the maximum value of the force generated by OHC, *x*_1_ and *x*_2_ are the displacement amplitudes at which the set points of transition between states are found, and *a*_1_ and *a*_2_ are the displacement amplitude sensitivities of the transitions [17]. In the present model, since the force generated by OHC, *F*_OHC_, is set to be 0.0 nN when *x* is 0.0 nm, (17) transforms into

(18) FOHC,0x=Fmax11+ea1x1−x1+ea2x2−x−11+ea1x11+ea2x2.

**Figure 6:**
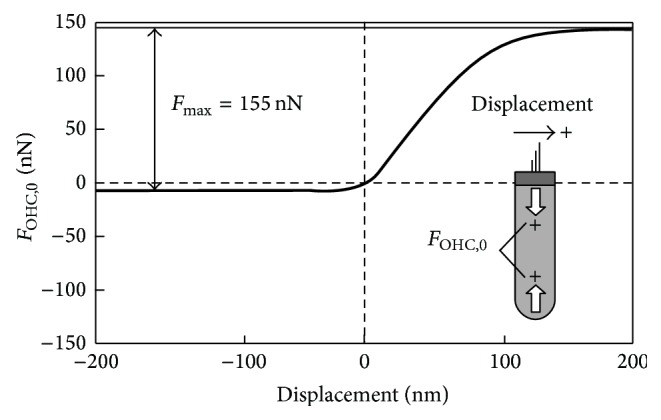
Estimated relationship between the force generated by the OHC and the displacement amplitude of the tip of its stereocilia. The maximum value of the force generated by the OHC *F*_max_ is estimated to be 155 nN to obtain a gain of the BM displacement amplitude (*x*_BM_) of 28 dB at 16 kHz with a stimulus intensity of 20 dB SPL. Positive displacement and positive force are defined as shown in the schematic of the OHC.

According to the experiment reported by Géléoc et al. [17], *x*_1_, *x*_2_, *a*_1_, and *a*_2_ are determined to be 8.2 nm, 49.0 nm, 0.092 nm^−1^, and 0.038 nm^−1^, respectively. In (18), *F*_max_ is estimated to be 155 nN to obtain a gain of 28.0 dB in the displacement amplitude of the BM caused by the force generation by the OHC at the CF of 16 kHz and 20 dB SPL, which is close to the previously obtained experimental result, 30.0 dB [19].  Figure 6 shows the relationship derived from (18). Deflection of stereocilia toward the tallest hair, which induces depolarization of membrane potential and contraction of the OHC, is defined as a positive displacement, as shown in the schematic in this figure.

In a previous numerical study, it was found that the phase delay of the force generation by the OHC motility relative to the deflection of its hair bundle is possibly within the range of 45 degrees to 180 degrees. Otherwise, the vibration frequency of the BM is not synchronized with the stimulus frequency [10]. In accordance with previously reported information about the phase delay of the force generated by the OHC motility relative to the displacement of its hair bundle, such delay is assumed to be 90 degrees in the present study. Since one cycle of the vibration is divided into 32 time steps in this study, the phase delay of 90 degrees becomes 8 steps. The force generated by OHC at time step* n F*_OHC_^*n*^ is given by

(19) $$\begin{array}{c} {F_{OHC}}^{n}={F_{OHC,0}}^{n-8}. \end{array}$$

The force calculated from (19) was applied to the top and bottom of the OHC model so that it would act in parallel to the longitudinal axis of the cell.

### 2.3. Numerical Procedure

In the previous report [1], as a structural model (OC) and fluid models (lymph fluid in the SV, SM, ST, and subtectorial space) were constructed separately, the fluid-structure interactions between the OC model and the lymph fluid models were considered by applying a staggered approach.

**Figure 7:**
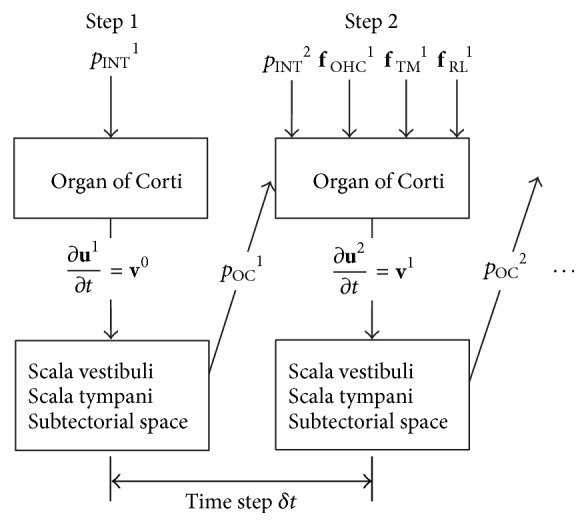
Schematic of the fluid-structure interaction using a staggered approach. In Step 1, initial pressure *p*_INT_^1^ is applied to the model of the OC and the velocity vector of the OC ∂**u** ^1^/∂*t* is obtained. Then, applying this obtained velocity vector ∂**u** ^1^/∂*t* to the SV, ST, and subtectorial space as a fluid velocity vector **v** ^0^ over a fluid-structure interface, the pressure *p*_OC_^1^ in the SV, ST, and subtectorial space caused by the movement of the OC is obtained. Next, the forces exerted on the TM and RL caused by the shear motion between the TM and RL **f**_TM_ and **f**_RL_ are obtained by (15) and (16). In Step 2, the displacement amplitude of the OHC stereocilia is calculated. Substituting this obtained value into (18), *F*_OHC,0_^1^ is determined, and thus *F*_OHC_^1^ is determined by (19). *F*_OHC_^1^ is then applied to nodes in the OC model corresponding to the apical and basal ends of the OHC as the force vector **f**_OHC_^1^. The previously obtained pressure *p*_OC_^1^ and the initial pressure *p*_INT_^2^ in time step 2 and the force vectors **f**_OHC_^1^, **f**_TM_^1^, and **f**_RL_^1^ are applied to the OC. By repeating the above procedure, the displacement amplitudes of the OC and those of the stereocilia at a specific stimulus frequency and a specific stimulus intensity are obtained.

The numerical procedure is as follows: First, the stimulus frequency and intensity are fixed to specific values. As shown in Figure 7, in the first step, initial pressure *p*_INT_^1^ is applied to the OC model (hatched area shown in Figure 3) and the velocity vector of the OC ∂**u** ^1^/∂*t* is obtained. Then, applying this obtained velocity vector ∂**u** ^1^/∂*t* to the SV, ST, and subtectorial space models as a fluid velocity vector **v** ^0^ over a fluid-structure interface, the pressure *p*_OC_^1^ in the SV, ST, and subtectorial space caused by the movement of the OC is obtained. The force vectors **f**_TM_^1^ and **f**_RL_^1^ caused by the shear motion between the TM and the RL are given by substituting the relative velocity vector *V*_relative_ of the TM in relation to the RL into (15) and (16), respectively. In the second step, the displacement amplitude of the tip of the OHC stereocilia is calculated. Substituting this obtained value into (18), *F*_OHC,0_^1^ is determined, and thus the OHC force *F*_OHC_^1^ is determined by (19). Then, *F*_OHC_^1^ is applied to nodes in the OC model corresponding to the apical and basal end of the OHC as the force vector **f**_OHC_^1^. The previously obtained pressure *p*_OC_^1^ and the initial pressure *p*_INT_^2^ in time step 2 and the force vectors **f**_OHC_^1^, **f**_TM_^1^, and **f**_RL_^1^ are applied to the OC. By repeating the above procedure, the displacement amplitudes of the OC and those of the stereocilia at a specific stimulus frequency and a specific stimulus intensity are obtained.

In the present study, the dynamic behavior of the OC, including the force generation by the OHC, was simulated for stimulus intensities from 20 dB SPL to 100 dB SPL.

### 2.4. Gain of the Basilar Membrane Vibration Caused by Cochlear Amplification

To confirm that the cochlear amplification of the model is appropriate, the gain of the displacement amplitude of the BM beneath the foot of the outer pillar cell (point shown in Figure 3), *x*_BM_, was compared to those reported in the previous experiment [19].

### 2.5. Functional Changes in OHC

To determine the effects of the functional loss of the OHC on the cochlear amplification, functional loss of OHCs is simulated by setting *F*_max_ in (18) to 0 nN. The conditions of the three rows of OHCs, that is, OHC-1, OHC-2, and OHC-3, as shown in Figure 3, are represented by A (active) or P (passive); that is, the former and the latter represent functional and dysfunctional OHCs, respectively. For example, the condition AAP means that OHC-1 and OHC-2 are functional but that OHC-3 is not.

In the present study, to evaluate the dynamic behavior of the OC, change in *x*_BM_ was analyzed in the following two cases: in Case 1, OC conditions were changed from AAA to PAA, PPA, and PPP, representing that OHCs lost their function from OHC-1 to OHC-3 as in injury caused by noise exposure [20]; in Case 2, OC conditions were changed from AAA to AAP, APP, and PPP, representing that OHCs lost their function from OHC-3 to OHC-1 as in injury caused by ototoxic acid [21], aging [22], transient cochlear ischemia [23], and so forth. The stimulus frequency was 16 kHz and stimulus intensities were 60 and 100 dB SPL in both cases.

Effects of the increase and decrease in the OHC maximum force on cochlear amplification were also analyzed since such changes in OHC force have been reported in previous studies. According to a previous study, the OHC function might be changed by 30% due to mutation in the prestin gene [24]. Thus, to determine the effects of such changes in the OHC function on cochlear amplification, the gains of *x*_BM_ when the OHC maximum force, *F*_max_ in (18), was set to 100 nN (30% decrease from 155 nN), 155 nN, 160 nN, 165 nN, 175 nN, 185 nN, and 200 nN (30% increase from 155 nN) were calculated.

In addition, to determine the OHC minimum force at which the system becomes unstable, changes in the gains of *x*_BM_ with a decrease of the OHC force from 200 nN to 0 nN at different stimulus intensities were calculated.

## 3. Results

### 3.1. Cochlear Amplification Caused by OHC Force

**Figure 8:**
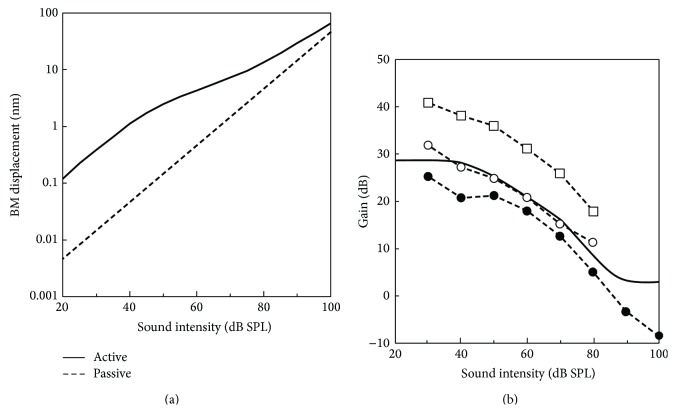
The I/O function of *x*_BM_ at the CF of 16 kHz. The input sound pressure ranged from 20 to 100 dB SPL. (a) *x*_BM_ in the active (i.e., the OHCs are functional with maximum forces of 155 nN) and passive states. (b) Gain of *x*_BM_ in the active state relative to that in the passive state. Calculated result, solid line; experimental result by Ren and Nuttall [19], dotted line with closed circles; experimental results by Overstreet et al. [25], dotted lines with open circles and squares.

Figure 8(a) shows the input/output (I/O) function of the BM displacement *x*_BM_ in the active state; that is, the OHCs are functional with maximum forces of 155 nN, and the passive state at the CF of 16 kHz with stimulus intensities ranging from 20 dB SPL to 100 dB SPL.  Figure 8(b) shows the gain of *x*_BM_ in the active state relative to that in the passive state at the CF of 16 kHz. As shown in Figure 8(a), the I/O curve in the active state (solid line) showed a nonlinear response; that is, the BM displacement *x*_BM_ showed a slight nonlinear increase below 50 dB SPL while it was about 10 times larger than that calculated from the passive state (dotted line), and such increase was then suppressed at around 50–80 dB SPL. At over 80 dB SPL, the BM displacement *x*_BM_ started to increase again while it still showed a slight nonlinearity. In Figure 8(b), the solid line shows the gain of *x*_BM_ calculated from (a). Its tendency is similar to that obtained from previous experimental measurements for each stimulus intensity (Ren and Nuttall [19]; Overstreet et al. [25]; dotted lines); that is, it decreases with an increase in the stimulus intensity.

### 3.2. Effects of OHC Functional Loss on Cochlear Amplification

**Figure 9:**
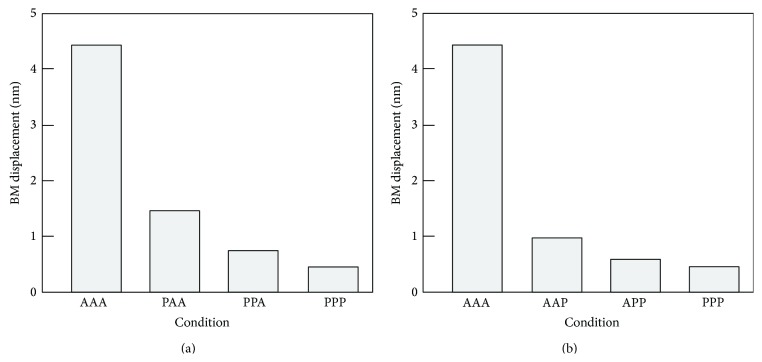
Change in *x*_BM_ caused by functional loss of OHCs at 60 dB SPL. (a) Case 1: functional loss occurred from OHC-1 to OHC-3. (b) Case 2: functional loss occurred from OHC-3 to OHC-1.

Figure 9(a) shows changes of *x*_BM_ in Case 1; that is, the OC condition changed from AAA to AAP, APP, and PPP with the stimulus intensity of 60 dB SPL. According to this figure, *x*_BM_ were significantly decreased when the first functional loss occurred and amount of reductions became small as the OHCs lost their function row by row.  Figure 9(b) shows changes of *x*_BM_ in Case 2; that is, the OC condition changed from AAA to PAA, PPA, and PPP. Its tendency in the reduction was similar to that of (a); that is, *x*_BM_ decreased most when the first functional loss occurred and amount of decrease became smaller row by row. Comparison of the conditions of PAA in (a) and AAP in (b) indicates that OHC-3 seems to have a 1.2 times greater effects on the cochlear amplification than OHC-1. The numerical data are summarized in Table 2.

**Figure 10:**
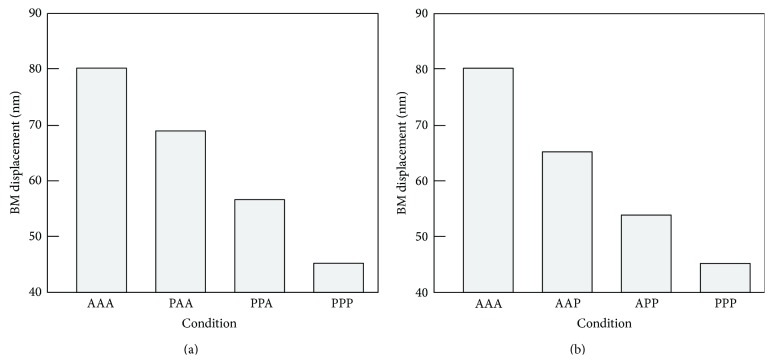
Change in *x*_BM_ caused by functional loss of OHCs at 100 dB SPL. (a) Case 1: functional loss occurred from OHC-1 to OHC-3. (b) Case 2: functional loss occurred from OHC-3 to OHC-1.

At 100 dB SPL, on the other hand, a different tendency was obtained. Figures 10(a) and 10(b) show changes of *x*_BM_ in Cases 1 and 2, respectively. The BM displacement linearly decreased with an increase in the number of dysfunctional OHCs. The numerical data are summarized in Table 3.

### 3.3. Effects of Increase and Decrease of OHC Force on Cochlear Amplification

**Figure 11:**
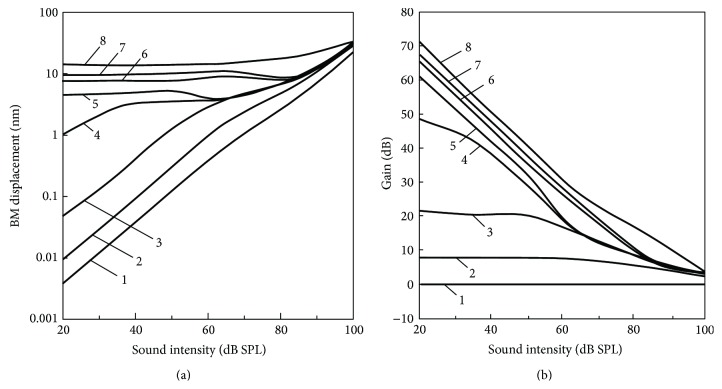
The I/O function of *x*_BM_ at the CF of 16 kHz with different degrees of force generated by OHC. (a) The BM displacement. 1: the maximum OHC force was 0 nN; 2: 100 nN; 3: 155 nN; 4: 160 nN; 5: 165 nN; 6: 175 nN; 7: 185 nN; 8: 200 nN. (b) Gain of the BM displacement amplitude.

Figure 11(a) shows the input/output (I/O) function of *x*_BM_ with *F*_max_ of 0, 100, 155, 160, 165, 175, 185, and 200 nN at the CF of 16 kHz with stimulus intensities ranging from 20 dB SPL to 100 dB SPL, and Figure 11(b) shows the gain of *x*_BM_ in the active state relative to that in the passive state at the CF of 16 kHz. As shown in this figure, when *F*_max_ was set to 200 nN, *x*_BM_ was too greatly amplified by the cochlear amplification and reached around 20 *μ*m regardless of the sound intensity at around 20–80 dB SPL. Thus, the gain of *x*_BM_ was over 70 dB at around 20 dB SPL. On the other hand, when *F*_max_ was set to 100 nN, the cochlear amplification was significantly small and the gain of *x*_BM_ was less than 10 dB at maximum.

**Figure 12:**
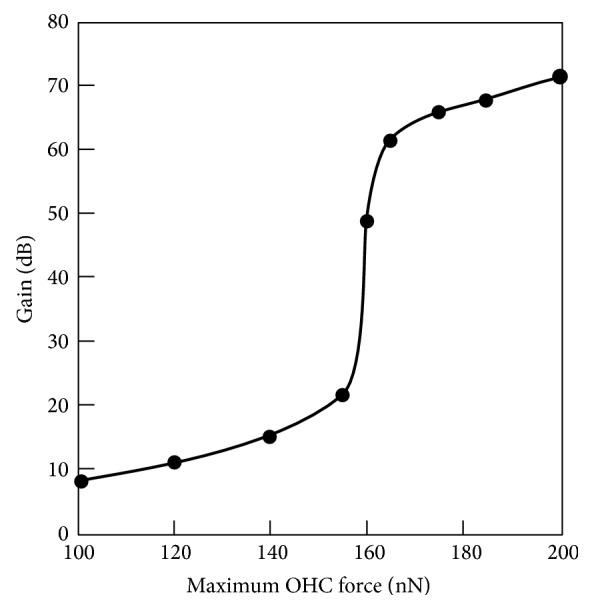
The gain of *x*_BM_ as a function of *F*_max_ when the stimulus intensity was 20 dB SPL.

The gain of *x*_BM_ as a function of *F*_max_ when the stimulus intensity was 20 dB SPL is shown in Figure 12. As shown in this figure, the gain of *x*_BM_ rapidly increased at around 160 nN of maximum OHC force and tended to be saturated with an increase of force.

**Figure 13:**
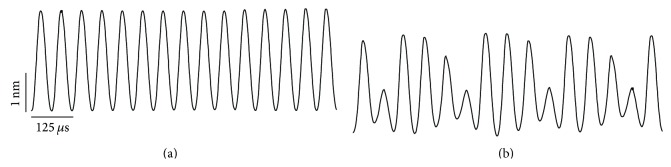
Time courses of the BM displacement with different OHC maximum forces when the stimulus intensity was 65 dB SPL. (a) The OHC maximum force was 115 nN. (b) OHC maximum force was 100 nN.

Figure 13 shows the time courses of the BM displacement with different OHC maximum forces when the stimulus intensity was 65 dB SPL. Although the system remained stable when the OHC maximum force was 115 nN, as shown in Figure 13(a), this was not the case when such force was 100 nN, as shown in Figure 13(b). When the OHC maximum force and the stimulus intensity became less than 100 nN and smaller than or equal to 65 dB SPL, respectively, the system was no longer stable.

## 4. Discussion

### 4.1. Validation of the Model: Cochlear Amplification

In Figure 8(b), the amplitudes of the gain obtained by the calculation were slightly larger than those obtained by experiment [19] and slightly lower than the other experimental result [25]. This is thought to have been caused by the difference in the experimental conditions; that is, although the calculation was done with the model in which the CF was 16 kHz, the experiment was done at CFs of 14 and 35 kHz, respectively. Since it is known that the basal region of the cochlea tends to have a larger gain of cochlear amplification relative to the apical region, the gain at the CF of 16 kHz is larger than that at the CF of 14 kHz and lower than that at the CF of 35 kHz.

When the stimulus intensity was over 80 dB SPL, the experimental result by Ren and Nuttall [19] showed a negative gain. However, in the present study, such attenuation was not observed in the numerical result. Although it is difficult to specify the reason for this discrepancy, one possibility is the phase delay of the OHC forces relative to the bending motion of the stereocilia. In an experiment in chinchilla, such delay was changed by the sound stimulus [26]. In the present model, however, the phase delay was fixed at 90 degrees regardless of the sound stimulus. To explore possible clues for attenuation of the BM gain caused by the OHC forces, taking the effects of the changes in phase delay of the OHC force on the cochlear amplification at a high sound stimulus into account is therefore thought to be useful.

### 4.2. Reduction in Cochlear Amplification by OHC Functional Loss

In the present study, *x*_BM_ was significantly decreased when only one of the OHCs became dysfunctional at 60 dB SPL. A previous experiment using noise exposure to the basal turn of rat cochlea, in which OHCs lost their function from OHC-1 to OHC-3, showed that the compound action potential (CAP) threshold shift was around 38, 70, and 72 dB when the OHC loss was 30, 80, and 100%, respectively [20]. According to those results, the relationship between the CAP threshold shift and OHC loss was not proportional; that is, the initial 30% OHC loss seems to have a greater effect on the CAP threshold shift than OHC loss from 30% to 80% and that from 80% to 100%. Since the 33% OHC loss in the experimental results is thought to correspond to dysfunction of a single row of OHCs in the present calculated results and since the CAP threshold shift is thought to be correlated to changes in the displacement amplitude of the OC, it can be said that results obtained by the calculation (Figure 9(a)) showed the same tendency as those obtained by the experiments; that is, the initial dysfunction of a single row of OHCs has the greater effect on the cochlear amplification than second and third dysfunctions of a single row of OHCs.

Another experiment using styrene, which was orally dosed, showed CAP threshold shifts of 8, 34, and 32 dB with 35, 70, and 100% OHC losses from OHC-3 to OHC-1, respectively [21]. In this case, OHC loss from 35% to 70% had greater effects on the cochlear amplification than that of the first 35% or that from 70% to 100%, a tendency different from that shown by our numerical results (Figure 9(b)). Although the reason for such difference is unclear, the effects of an oral dose of the styrene on the cochlear function should be considered. Unlike the mechanism of noise-induced hair cell loss, the mechanism of styrene-induced hair cell loss must involve a much more complicated pathway; that is, the styrene probably affects not only OHCs but also other parts of the animal.

**Figure 14:**
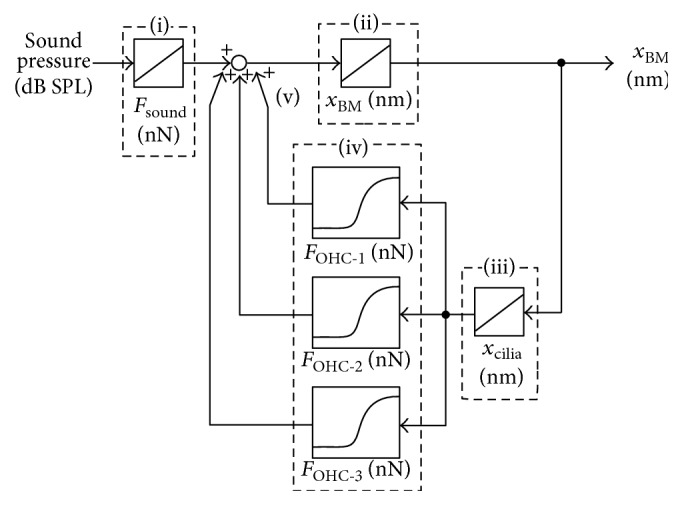
Block diagram of the OC. Force caused by sound stimulus *F*_sound_ is applied to the OC (i), and *x*_BM_ is then calculated (ii), leading to *x*_cilia_ (iii). The forces generated by OHCs are determined by *x*_cilia_ based on the force-displacement map, shown in Figure 6 (iv). OHC forces are applied to the OC in combination with *F*_sound_ (v), thus being known as a positive feedback system.

From the viewpoint of mechanics, the mechanisms of significant decreases in the BM displacement caused by functional loss of OHCs can be explained by the positive feedback system of the OC using a block diagram, as shown in Figure 14. In this block diagram, dynamic behavior of the OC is simplified as follows: First, the force due to the sound pressure *F*_sound_ is applied to the OC model ((i), Figure 14). Then, *x*_BM_ is calculated (ii), leading to *x*_cilia_ (iii). OHC forces are determined by *x*_cilia_ based on the force-displacement map shown in Figure 6 (iv). OHC forces are applied to the OC model in combination with *F*_sound_ at the next step (v). Therefore, *x*_BM_ is increased and thereby *x*_cilia_ is increased, leading to increases in OHC forces themselves.

Table 2 shows *x*_BM_, *x*_cilia_, and OHC forces *F*_OHC-1_, *F*_OHC-2_, and *F*_OHC-3_ in each OHC condition at 60 dB SPL. In this study, when one of the OHCs lost its function, *x*_BM_ and thereby *x*_cilia_ decreased. Since OHC forces were calculated by *x*_cilia_ shown in Figure 6, total force generated by the remaining OHCs decreased. Since such OHC force was positively feedbacked and triggered the vibration of the BM, *x*_BM_ decreased and thereby *x*_cilia_ decreased more, resulting in a significant decrease in *x*_BM_ when one of OHCs lost its function. At 100 dB SPL, on the other hand, *x*_BM_ decreased almost linearly when OHCs became dysfunctional row by row. In this case, due to high input sound pressure, the BM displacement *x*_BM_ exceeds 45 nm even if all OHCs are dysfunctional, being about 100 times greater than that calculated at 60 dB SPL (Tables 2 and 3). The maximum displacement of the stereocilia *x*_cilia_ therefore reaches from about 150 nm to about 200 nm, with the result that OHCs generate near maximum forces regardless the OHC conditions (Table 3). Since OHC force amplitudes did not decrease despite the functional loss of OHCs, *x*_BM_ decreased linearly when OHCs lost their function row by row, as shown in Figures 10(a) and 10(b). These data suggest that all three rows of OHCs are indispensable for maintaining normal cochlear amplification.

### 4.3. Effects of Changes in OHC Force on the Cochlear Amplification

As shown in Figures 11(a) and 11(b), when *F*_max_ was set to 200 nN, *x*_BM_ was too greatly amplified and reached around 20 *μ*m regardless of the sound intensity at around 20–80 dB SPL. Such tendency started to appear when *F*_max_ reached a level 160 nN. This result means that it is impossible to distinguish the sound intensity between 20 and 80 dB SPL from the value of *x*_BM_, suggesting that the enhancement of the OHC function caused by mutation of the prestin gene may possibly spoil the intensity selectivity of the OC.

As shown in Figures 11 and 13, the cochlear amplification was significantly attenuated by a decrease in the OHC maximum force and became unstable when such force was smaller than 100 nN. Taking these results into consideration, it can be said that the cochlear amplification caused by the function of the OHCs is well regulated under natural conditions. Thus, neither an increase nor a decrease in the OHC maximum force is good for our hearing.

## 5. Conclusions

In the present study, an OC model including cochlear amplification was constructed. The displacement of the BM *x*_BM_ was significantly decreased when only a single row of OHCs lost its function, suggesting that all three rows of OHCs are required for cochlear amplification.

## Figures and Tables

**Figure 15:**
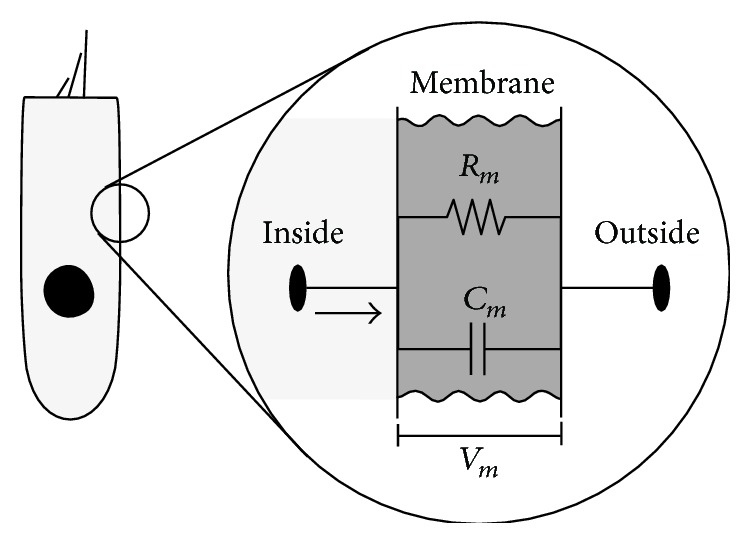
Electrical circuit model of the cell membrane. *R*_*m*_ and *C*_*m*_ are the membrane resistance and membrane capacitance, respectively. *I*_*m*_ is the current across the membrane and *V*_*m*_ is the membrane potential. The membrane impedance, which acts as the low-pass filter, consists of a parallel connection of *R*_*m*_ and *C*_*m*_.

**Table 1:** Young's modulus and Poisson's ratio assigned to the OC model.

	Young's modulus (Pa)	Poisson's ratio
BM (fibrous layer)	1.0 × 10^7^	0.30
BM (hyaline matrix)	3.0 × 10^4^	0.49
Deiters' cell	1.0 × 10^7^	0.30
Hensen's cell	5.0 × 10^3^	0.49
IHC	1.0 × 10^4^	0.49
Kimura's membrane	1.0 × 10^6^	0.30
Osseous spiral lamina	2.0 × 10^10^	0.30
OHC	1.0 × 10^4^	0.49
Phalanx	1.0 × 10^7^	0.30
Pillar cells	1.0 × 10^9^	0.30
RL	1.0 × 10^9^	0.30
Stereocilia	1.0 × 10^7^	0.30
TM	3.0 × 10^4^	0.49

**Table 2:** Force generated by each OHC, and displacement amplitudes of the stereocilia and the BM at 60 dB SPL.

Case	Condition	*F*_OHC-1_ [nN]	*F*_OHC-2_ [nN]	*F*_OHC-3_ [nN]	*x*_BM_ [nm]	*x*_cilia_ [nm]
1, 2	AAA	13.4	14.2	15.0	4.42	14.3
1	PAA	0.0	3.6	3.8	1.47	4.1
1	PPA	0.0	0.0	2.0	0.72	2.4
2	AAP	2.3	2.4	0.0	0.97	3.2
2	APP	1.3	0.0	0.0	0.57	1.9
1, 2	PPP	0.0	0.0	0.0	0.47	1.5

**Table 3:** Force generated by each OHC, and displacement amplitudes of the stereocilia and the BM at 100 dB SPL.

Case	Condition	*F*_OHC-1_ [nN]	*F*_OHC-2_ [nN]	*F*_OHC-3_ [nN]	*x*_BM_ [nm]	*x*_cilia_ [nm]
1, 2	AAA	147.4	147.4	147.4	80.05	212.8
1	PAA	0.0	147.0	147.2	72.36	191.5
1	PPA	0.0	0.0	146.3	53.35	168.9
2	AAP	146.7	146.9	0.0	65.10	182.7
2	APP	145.4	0.0	0.0	49.58	159.9
1, 2	PPP	0.0	0.0	0.0	45.65	148.4

## Appendix

### Low-Pass Filter Properties of the Cell Membrane

The electrical impedance of the membrane *Z*_*m*_ is represented as the resistance *R*_*m*_ and capacitance *C*_*m*_ in parallel as shown in Figure 15. When the receptor current *I*_*m*_ flows across the cell membrane, the receptor potential *V*_*m*_ can be described as follows:

(A.1) $$\begin{array}{c} V_{m}=Z_{m}I_{m}, \end{array}$$

where

(A.2) $$\begin{array}{c} Z_{m}=\frac{R_{m}}{1+j\omega R_{m}C_{m}}. \end{array}$$

In (A.2), *j* = (−1)^1/2^ and *ω* is the angular frequency of the receptor current expressed by *ω* = 2*πf*, where *f* is the frequency. This angular frequency *ω* is the same as that of the pure tone applied to the ear canal because the receptor current is caused by the deflection of the OHC hair bundle which follows the OC vibration excited by the pure tone in the ear canal. In this case, the magnitude |*V*_*m*_| and the phase *θ* of the membrane potential relative to the current *I*_*m*_ across the membrane are given by

(A.3) Vm=Rm1+ω/ω02Im,

(A.4) θ=−arctan⁡ωω0,

where *ω*_0_ = 1/(*R*_*m*_ *C*_*m*_) represents the cut-off frequency. Equations (A.3) and (A.4) show that the magnitude |*V*_*m*_| is small and the phase *θ* is −90 degrees when the angular frequency of the receptor current *ω* is well above the cut-off frequency *ω*_0_. Therefore, it is clear that the cell membrane possesses low-pass filter properties.
